# Comparison of Drive Counts and Mark-Resight As Methods of Population Size Estimation of Highly Dense Sika Deer (*Cervus nippon*) Populations

**DOI:** 10.1371/journal.pone.0164345

**Published:** 2016-10-06

**Authors:** Kazutaka Takeshita, Takashi Ikeda, Hiroshi Takahashi, Tsuyoshi Yoshida, Hiromasa Igota, Yukiko Matsuura, Koichi Kaji

**Affiliations:** 1 Laboratory of Wildlife Management, Tokyo University of Agriculture and Technology, Fuchu, Tokyo, Japan; 2 Kansai Research Center, Forestry and Forest Products Research Institute, Kyoto, Kyoto, Japan; 3 Department of Environmental and Symbiotic Science, Rakuno Gakuen University, Ebetsu, Hokkaido, Japan; 4 Hokkaido Research Center, Forestry and Forest Products Research Institute, Sapporo, Hokkaido, Japan; Centre for Cellular and Molecular Biology, INDIA

## Abstract

Assessing temporal changes in abundance indices is an important issue in the management of large herbivore populations. The drive counts method has been frequently used as a deer abundance index in mountainous regions. However, despite an inherent risk for observation errors in drive counts, which increase with deer density, evaluations of the utility of drive counts at a high deer density remain scarce. We compared the drive counts and mark-resight (MR) methods in the evaluation of a highly dense sika deer population (MR estimates ranged between 11 and 53 individuals/km^2^) on Nakanoshima Island, Hokkaido, Japan, between 1999 and 2006. This deer population experienced two large reductions in density; approximately 200 animals in total were taken from the population through a large-scale population removal and a separate winter mass mortality event. Although the drive counts tracked temporal changes in deer abundance on the island, they overestimated the counts for all years in comparison to the MR method. Increased overestimation in drive count estimates after the winter mass mortality event may be due to a double count derived from increased deer movement and recovery of body condition secondary to the mitigation of density-dependent food limitations. Drive counts are unreliable because they are affected by unfavorable factors such as bad weather, and they are cost-prohibitive to repeat, which precludes the calculation of confidence intervals. Therefore, the use of drive counts to infer the deer abundance needs to be reconsidered.

## Introduction

Deer overabundance has severe and negative effects on biodiversity and natural ecosystems [1], and the appropriate management of deer populations is an urgent global issue. In the past century, the monitoring of deer density played a fundamental role in the decision-making process and assessment of deer management programs. However, there are few methods that provide accurate estimates of population size, and these approaches are often constrained by their methodological characteristics when incorporated in a practical management program (for example, the capture-mark-resighting method, which can provide accurate estimates of population size [2–5], can hardly be applied to a large spatial distribution). Moreover, absolute population size by itself does not provide information on the relationship between the deer population and surrounding habitat. Therefore, Morellet et al. [6] proposed managing populations of large herbivores and monitoring population-habitat systems through a set of indicators of animal performance [7], herbivore impact on habitat [8,9], and population abundance [4,10]. Temporal changes in the abundance of large herbivores have been examined using abundance indices calculated by the fecal pellet group count method [11], spotlighting method [4,12,13], thermal imagery method [14], aerial survey [15], and hunting statistics (seen per unit effort [SPUE], catch per unit effort [CPUE] [16–18]). However, the applicability of these methods depends on the habitat features of the management area. For example, aerial survey [15] is unsuited for deer inhabiting thickly timbered conifer forests, and its economic feasibility and availability of an experienced pilot vary greatly by country. Consequently, in accordance with time, personnel, funds, climate, topography, and vegetative features of the management area, wildlife managers must choose the most suitable method amongst various abundance indices. Furthermore, when managers choose abundance indices, potential biases and their effect on population estimates must be considered [19].

In Japan, the overabundance of sika deer is a major concern for wildlife managers and foresters. High deer densities that exceeded 50 individuals/km^2^ were observed in several local populations in national parks (e.g., Tanzawa Mountains of Kanagawa Prefecture [20]). Single yearly ground counts such as drive counts [21] are a frequently used method to estimate sika deer densities in Japan, a region with steep terrain and bushy forests. Drive counts have been frequently used in enclosed habitats and mountainous regions with well-defined topographic boundaries. For forest-dwelling red deer (*Cervus elaphus*) and roe deer (*Capreolus capreolus*) populations, based on computer simulation, Borkowski et al. [22] suggested that deer density is an important factor influencing the accuracy of drive counts. For example, these counts may be more reliable with higher deer densities (more than approximately 5–7 individuals/km^2^) as long as a lower level of accuracy (within 20% or more) is acceptable, but drive count would not be suitable if a high degree of accuracy (e.g., within 10% of the true value) is required, regardless of an increasing deer density [22]. However, the authors simulated the effect of increasing deer density on the accuracy of drive counts in populations of only up to 22 individuals/km^2^, and therefore the merit of this method at higher population densities remains unclear. Under high-density conditions, observation error for drive counts would increase with a rise in population density [23]. In this way, implementing drive counts at higher densities is challenging.

The population of sika deer on Nakanoshima Island, which is located in the center of Lake Toya, in the southwestern part of the Hokkaido Prefecture, Japan, was established after three sika deer were introduced in the middle of the last century [24]. Hunting was prohibited, and the population grew steadily. Deer abundance dropped precipitously after a large-scale deer removal in 2001, when 102 deer were artificially transplanted from the island in March 2001, and a mass mortality event in the winter of 2003–2004 [25], as evidenced by the collection of over 100 carcasses of deer that died naturally from a food shortage during that period. These large population reductions provided opportunities to test the utility of abundance indices [12]. In the present study, we compared drive counts with the reference mark-resight (hereafter, MR) method. The aim of this study was to evaluate the utility of drive count estimates as an abundance index under conditions of extremely high deer density (MR estimates between 11 and 53 individuals/km^2^). In particular, we focused on whether drive counts showed a consistent trend with MR and were able to reflect the large population reductions that occurred in 2001 and 2004.

## Materials and Methods

### Study area

**Fig 1:**
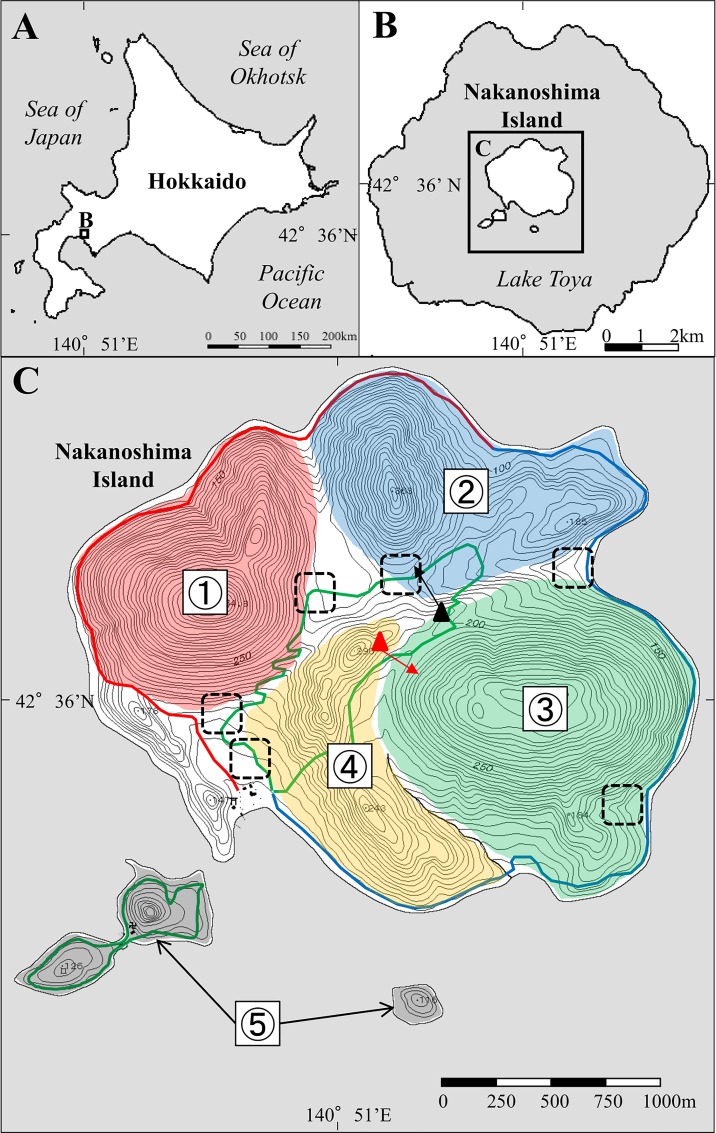
Map of Nakanoshima Island, Lake Toya, Hokkaido, Japan, showing three fixed routes followed for the mark-resight (MR) method, five blocks surveyed by the drive counts method, and main deer capture sites. From 2002, the outer course was divided into east and west routes to reduce survey time, and the resulting three fixed routes are indicated by red, blue, and green lines. The five blocks for drive counts are shown as red, blue, green, yellow, and black polygons, and the positions of the vantage point observers in the drive count surveys are indicated by triangles (the black triangle and black arrow indicate the position and direction of the vantage point observer conducting the survey of blocks 1 and 2, and the red triangle and red arrow indicate the position and direction of the vantage point observer conducting the survey of blocks 3 and 4). Black dotted squares indicate main deer capture sites.

The study population consisted of a sika deer population located on Nakanoshima Island (42°36ʹ N, 140°51ʹ E, Fig 1). The island consists of a main island (4.978 km^2^) and two small islands (0.23 and 0.038 km^2^). There are three mountains rising from 80 to 450 m above sea level on the main island. The climate is characterized by warm summers and snowy winters. Average yearly precipitation between 1999 and 2006 (excluding 2000 because of incomplete data due to the eruption of Mt. Usu located to the south of Lake Toya) was 1035.3 mm, with a range of 912.0–1,185.0 mm, at the Toyakoonsen weather station [26] that is located 6 km southwest of the island. The average maximum snowfall in March between 1999 and 2006 was 37.8 cm, with a range of 24.7–59.9 cm, at the Toya Lake Station, Field Science Center for the Northern Biosphere, Hokkaido University, located 4 km west of the island. The majority of the island surface (91.8%) is covered by deciduous broad-leaved trees, including oak (*Quercus crispula*), castor-aralia (*Kalopanax pictus*), magnolia (*Magnolia obovata*), maple (*Acer mono*), and linden (*Tilia japonica*) [27,28]. Vegetation on the forest floor has declined secondary to deer browsing pressure, and young deciduous trees have been nearly eliminated because of bark stripping by deer [27]. Therefore, visibility on the ground was favorable between the fall and spring.

Three sika deer (one male and two females) were introduced to Nakanoshima Island in 1957, 1958, and 1965, respectively, from the Hidaka district, which is located in the southeastern part of Hokkaido [24]. The deer had no predators and hunting was prohibited. Immigration and emigration on Nakanoshima Island were assumed negligible because Lake Toya does not freeze and the island is situated 4 km off the lakeshore, and because none of the 141 radio-collared deer left the island by swimming during the study period. In March of 2001, 102 deer were artificially transplanted from the island. Deer carcasses were collected by organized intensive searches (40–80 persons per year), conducted in April and May, and incidentally (during March drive counts). Based on necropsies of the carcasses, we estimated that at least 100 deer died between the survey date of drive counts in 2003 and that in 2004.

### Capture and marking

To conduct a deer monitoring program on the island, we captured and tagged 127 deer using a modified alpine capture system [29] or dart gun between March 1992 and March 2001, and 128 deer by using a corral trap [30] or dart gun between April 2001 and March 2005. All captured deer were equipped with numbered plastic tags attached to both ears, and 141 of the 255 captured deer were equipped with radio collars. The corral trap used between April 2001 and March 2005 was 361 m in circumference and composed of a funnel-shaped corral with wire net, J-shaped pen, and darkroom made of plywood [30]; captured deer were driven into the pen and the darkroom. In all capture methods, captured deer were immobilized with xylazine-ketamine (xylazine: 2.0 mg/kg, ketamine: 6.0 mg/kg) [31] or medetomidine-ketamine mixture (medetomidine: 0.1 mg/kg, ketamine: 5.0 mg/kg) [32], which were administered intramuscularly by blowpipe or dart gun. Deer captures were conducted by several methods over various sites and seasons, with the exclusion of select steep mountainous areas (Fig 1). In the present study, we additionally used two marked deer that were captured in previous studies conducted in March 1982 and March 1988 [33]. Thus, 257 marked deer (127 females and 130 males) were available for the present study. These captures were conducted under the permission obtained for the present study from the Hokkaido Government according to “Wildlife Protection and Proper Hunting Act” (Ministry of the Environment), which covered the capture, sedation, tagging, and collaring of deer. Permission numbers of the Hokkaido Government permits are FY1991-No. 71, FY1993-No. 96, FY1994-No. 165, FY1995-No. 164, FY1997-No. 195, FY1998-No. 1 and No. 66, FY1999-No. 15–19, FY2000-No. 4, FY2001-No. 9, FY2002-No. 55, FY2003-No. 40, and FY2004-No. 107.

### Mark-resight method

We conducted 30 resighting surveys along two fixed routes [34] (inner and outer courses) in the spring (from mid-April to late-May) between 1999 and 2006 over 3–5 consecutive days (Table 1). The inner course of the main island covered one small island, and the outer course of the main island was divided into east and west side routes starting from 2002 to reduce survey time (Fig 1). In most surveys, each course was surveyed by one observer, and observations were conducted with 12 × 36, 12 × 50, or 15 × 50 image-stabilizing binoculars. Observation time, location, presence or absence of ear tags, and tag number, if visible, were recorded for each observed deer. The surveys were conducted from 0700 to 1000 h to reduce the influence of cruise boats and tourists on deer behavior. The birth season of sika deer in Hokkaido is mainly from late May to June [35]. However, because we did not observe newborn fawns during resighting surveys in May, we assumed that fawns were typically born in June on the island [34]. Thus, we also assumed that the spring study period for MR was not influenced by changes in deer density related to the birth of fawns.

**Table 1:** Survey date, observations within each primary sampling occasion (i.e., year) and number of survived marked deer tracked by radio collars in the sika deer population on Nakanoshima Island from 1999 to 2006.

			Observation number of marked deer			
Year	Survey date	Number of survived marked deer being tracked by radio-collar	Identified	Unidentified	Observation number of unmarked deer	Observation ratio of all marked deer to all observed deer
1999	April 16–19	1	48	4	180	0.22
2000	May 16–19	0	103	7	199	0.36
2001	May 14–18	25	74	12	231	0.27
2002	April 17–19	47	63	5	152	0.31
2003	May 12–14	69	76	16	175	0.34
2004	May 12–14	70	92	5	98	0.50
2005	May 23–25	69	37	4	21	0.66
2006	May 18–22	32	63	9	67	0.52

The exact time of death was confirmed for 167 of 257 marked deer and was based on a mortality sensor attached to the radio collar and/or the necropsy of the carcass (the number of tracked deer using radio collars between 1999 and 2006 is summarized in Table 1). The survival of 49 of 257 marked deer after the study period (after May 22, 2006) was then confirmed based on direct observation and/or radio telemetry. However, because we could not identify the exact time of death for 41 of the 257 marked deer, the number of available marked deer in the study period remained unknown. Therefore, population size was inferred from the number of observed marked and unmarked deer using the Zero-truncated Poisson-log Normal estimator (ZPNE) [36,37] implemented in MARK [38,39]. ZPNE is a robust choice when the exact number of marked animals is not known and when sampling may be with replacement within primary sampling intervals [36]. Moreover, ZPNE successfully incorporates individual heterogeneity of sighting probabilities and takes into account observations that are distinguished only as marked or unmarked (i.e., the presence of a plastic ear tag is confirmed but not its number). To assess population size, ZPNE requires the following assumptions: (1) demographic and geographic closure of the population; (2) no loss of marks; (3) no errors in distinguishing between surveyed marked and unmarked individuals; and (4) independently and identically distributed resighting probabilities for marked and unmarked individuals [36,40]. Additionally, ZPNE assumes that the sampling of marks was representative of the study population. When we estimated the deer population size, each of the observations conducted over 3–5 consecutive days was regarded as a secondary sampling occasion (i.e., observation data were pooled within each year); because we did not confirm any fresh deer carcasses on 3–5 consecutive resighting surveys in each year, and therefore, the number of deer can be assumed to be stable within each primary sampling occasion (i.e., year).

When using ZPNE to estimate the population, the number of available marked deer was set to zero (i.e., unknown) for all primary sampling occasions. In addition, it was anticipated that there would be differences in resighting probabilities α between sexes. Moreover, it was expected that survival probability φ between primary occasions *j* and *j* + 1 may show annual variation. Thus, we built a set of eight ZPNE models with different parametrizations of resighting probability α and survival probability φ to find the best fit to our dataset. Because we could not distinguish the sex of unmarked individuals on resighting surveys, we performed MR estimation on the deer population size using sex as an individual binary covariate (male = 1 and female = 0). This approach assumes that the sex ratios are similar between marked and unmarked individuals [13]. For the full model, we modeled resighting probability α as a function of year plus sex, and survival probability φ as a function of year. For all models, we allowed for annual variations in the number of unmarked individuals in the population *U* during primary occasion *j*, whereas individual heterogeneity σ during primary occasion *j*, and transition probabilities γ' and γ'' between primary occasions *j* and *j* + 1 were kept constant. We compared eight models using the Akaike information criterion corrected for small samples (AICc). We considered the model significantly different when the ΔAICc was greater than 2, and when the ΔAICc was less than 2, we kept the model with the least number of parameters [41]. Building ZPNE models was performed using R 3.1.2 [42] and package RMark [43] as an interface for program MARK.

### Drive counts

Between 1999 and 2006, we estimated deer population size by drive counts using two small outboard-motor boats and approximately 30 drivers in early March. In this season, visibility from motor boats on the lake was favorable because forest cover was scarce and the ground was covered with deep snow. The study area was divided into five blocks including the adjacent two small islands (Fig 1). In each block, drivers lined up along the edge of the mountain or ridge and moved across one block until they arrived at the lakefront. Deer were forced to move by drivers, and we tallied deer being counted by observers from a boat and/or deer being forced to pass through the driver’s line to avoid double counting [24]. Additionally, to avoid double counting caused by deer movement across each block, we set a vantage point observer to monitor the expected deer path (Fig 1). This survey was conducted from 0830 to 1500 h, when deer are inactive [44], during each year.

### Statistical analysis

In the present study, we performed a standard major axis regression to evaluate the relationship between drive count and MR estimates because both variables (the drive count estimates and MR estimates) contain observation error. In the model, the log-transformed MR estimates were set as the independent variable and the log-transformed drive count estimates as the dependent variable. The results of the standard major axis regression were corrected for the drive count estimates in 2001 based on the artificial removal conducted just after the survey date, because artificial removal could cause irregular bias in regression analysis. Standard major axis regression was performed in lmodel2 package [45] for R.

## Results

**Fig 2:**
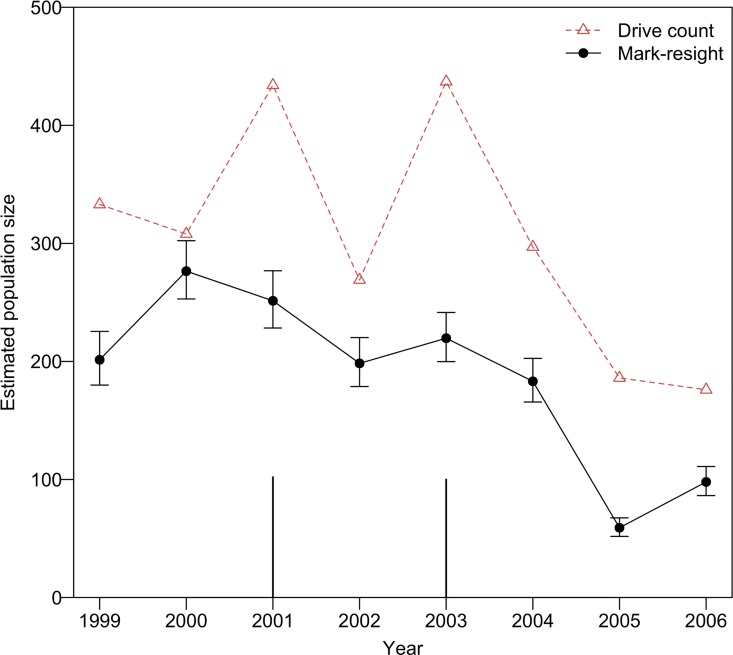
Temporal changes in population estimates obtained by drive counts and mark-resight (MR) methods in a sika deer population on Nakanoshima Island from 1999 to 2006. Open red triangles indicate drive counts estimates. Solid black circles and error bars indicate MR estimates and the corresponding 95% confidence intervals, respectively. The black bar in 2001 indicates the number of deer artificially transplanted from the island just after the drive counts in 2001 (102 individuals). The black bar in 2003 indicates the minimum number of deer that died of natural causes, whose estimated time of death was between the date of drive count survey in 2003 and that in 2004 (100 individuals).

**Fig 3:**
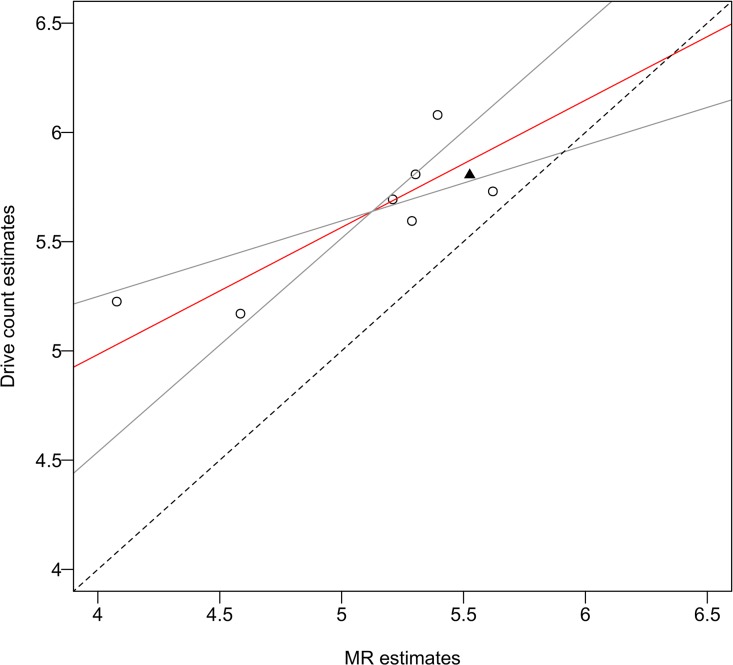
Relationship between drive count estimates and mark-resight (MR) estimates inferred from standard major axis regression in a sika deer population on Nakanoshima Island from 1999 to 2006. The solid red line indicates the regression line, and solid gray lines indicate the 95% confidence interval. The dashed black line indicates the equality line. The drive counts estimate in 2001 (solid triangle) was corrected for the artificial removal of deer before the regression analysis.

The results of model selection on a set of eight ZPNE models are shown in Table 2. We selected the model with resighting probability α as a function of sex and survival probability φ as a function of year as the best model, which had the lesser number of parameters among two models that had ΔAICc less than 2 (Table 2). On the best model, mean resighting probability α was 0.99, and mean survival probability φ between 1999 and 2006 was 0.94, with a range of 0.88–1.00. Annual population estimates obtained by the drive counts and MR methods appeared to be roughly parallel (Fig 2). After 2004, both drive count estimates and MR estimates showed a marked reduction in population size (Fig 2, Table 3). The drive counts method estimated larger values for all years compared to the MR method (Figs 2 and 3).

**Table 2:** Selection on the eight mark–resight models fitted to investigate the population size of sika deer on Nakanoshima Island from 1999 to 2006.

Model	AICc	ΔAICc	Akaike's weight	Number of parameters
**α(sex) σ(.) U(year) φ(year) γ'(.) γ''(.)**	**965.54**	**0.00**	**0.51**	**19**
α(sex + year) σ(.) U(year) φ(year) γ'(.) γ''(.)	967.35	1.81	0.21	26
α(sex) σ(.) U(year) φ(.) γ'(.) γ''(.)	967.91	2.37	0.16	13
α(.) σ(.) U(year) φ(year) γ'(.) γ''(.)	970.11	4.57	0.05	18
α(year) σ(.) U(year) φ(year) γ'(.) γ''(.)	970.55	5.01	0.04	25
α(sex + year) σ(.) U(year) φ(.) γ'(.) γ''(.)	971.98	6.44	0.02	20
α(.) σ(.) U(year) φ(.) γ'(.) γ''(.)	974.83	9.29	0.00	12
α(year) σ(.) U(year) φ(.) γ'(.) γ''(.)	975.21	9.67	0.00	19

The table shows model structure, Akaike’s information criterion corrected for small sample size (AICc), differences in AICc (ΔAICc) relative to the lowest value, the Akaike’s weights, and number of parameters for each model, and “+” indicates an additive effect. The selected model, for which ΔAICc is less than 2 with the least number of parameters, is presented in bold font.

**Table 3:** Population estimates using drive counts and mark-resight (MR) methods in a sika deer population on Nakanoshima Island from 1999 to 2006.

				95% CI^1^ of MR estimate			
Year	Survey date of MR	Population estimate of MR	SE^2^ of MR estimate	Lower bound	Upper bound	Survey date of drive count	Population estimate of drive count
1999	April 16–19	201	11.6	180	225	March 10	333
2000	May 16–19	276	12.6	253	302	March 16	308
2001	May 14–18	251	12.4	228	277	March 14	434
2002	April 17–19	198	10.6	179	220	March 6	269
2003	May 12–14	220	10.6	200	241	March 5	437
2004	May 12–14	183	9.4	166	203	March 2	297
2005	May 23–25	59	4	52	67	March 1	186
2006	May 18–22	98	6.3	86	111	March 2	176

^1^ SE indicates standard error.

^2^ CI indicates confidence interval.

The standard major axis regression showed a significant positive correlation between drive count estimates and MR estimates for the 8 years of study (Fig 3, Table 4). However, according to the model, the 95% confidence interval of the slope of the regression line did not include one, and the 95% confidence interval of the intercept of the regression line did not include zero (Table 4).

**Table 4:** Result of standard major axis regression.

			95% CI^1^ (Intercept)			95% CI^1^ (Slope)	
Dependent variable	Independent variable	Intercept	Lower bound	Upper bound	Slope	Lower bound	Upper bound
Drive count	Mark-resight	2.66	0.62	3.86	0.58	0.35	0.98

^1^CI indicates confidence interval.

## Discussion

In the present study, we evaluated the applicability of the drive counts method in inferring deer abundance index under high deer population density conditions (MR estimates between 11 and 53 individuals/km^2^) in reference to the MR method. The MR estimates identified two contrasting periods in deer abundance on the island: one in 1999–2004 and the other, 2005–2006. During the first period, MR estimates did not show large year-to-year variation, whereas the MR estimates had a marked reduction in the second period. Of 100 deer carcasses whose estimated time of death was between the date of the drive count survey in 2003 and that in 2004, 34 deer carcasses were found before the date of the resighting survey in 2003 (i.e., from March 5 to May 11; Table 3). Therefore, although MR estimates showed a reduction from 2003 to 2004, the reduction between May 2003 and May 2004 was not large. Furthermore, a marked reduction in MR estimates in the second period might be explained by a small-scale population removal during that period [46] and continued mass mortality in the late spring of 2004 (we collected 46 deer carcasses after the resighting survey in 2004). Because the true sika deer population size on the island is unknown, the comparison of the two estimates largely depended on the accuracy and precision of the MR method and two large population declines. Easily distinguishable numbered plastic ear tags (of seven colors and two sizes) effectively prevented errors in distinguishing between marked and unmarked individuals, but at the same time, the tags did not overtly attract observer attention because they do not reflect light. Likewise, secondary sampling occasions were conducted over a short timeframe on this geographically closed island. Moreover, the assumption that the sample of marks was representative of the study population was supported, because deer captures were conducted on various sites and seasons using several capture methods. However, the assumption of independent and identical distributed resighting probabilities (assumption 4, see “Mark-resight method” section in Materials and Methods) may be violated in the case of a social species such as sika deer. Nevertheless, the bias that was induced by non-independence of resightings could be considered consistent over the study period, and as this assumption should only affect the precision of the estimate (and not its accuracy), we assumed that we could evaluate the trends and consistencies in population size of the two focal methods.

Similar to the MR estimates, the drive counts estimates also showed marked reductions after 2004 and later. In contrast, the drive counts showed large year-to-year variation, especially around 2002. In 2001, there was a large difference in population estimates between the drive counts (434 individuals) and MR (251 individuals with 95% CI of 228–277) methods (Table 3). Although the difference between both estimates was reduced when considering the 102 deer that were removed just after the drive counts in 2001, the drive counts method still overestimated the population size. Moreover, there was a large fluctuation in drive counts estimates from 2002 to 2003, which was probably derived from poor visibility resulting from fog and little snow ground cover during the 2002 drive count survey. Consequently, although drive counts reflected the large population reductions in 2001 and 2004, the drive counts estimates were greater than the MR estimates for all years (Figs 2 and 3). The difference between the drive counts and MR estimates may then be derived from the difference in survey seasons: drive counts were conducted in early March, whereas MR surveys were conducted in April or May. Deer density may continue to decrease to some extent from March to May (i.e., late winter to early spring) because of malnutrition. However, the overestimation of drive counts decreases with an increase in density (after 2004; Fig 3, Table 4). If the difference between both estimates were caused by malnutrition, then it would be anticipated that the difference would become larger as density increased. Therefore, factors other than the difference in survey season were likely contributing to the overestimation of drive counts estimates under conditions of temporal reduction in deer density from extremely high deer density. For example, double counting could result from increased deer movement as the deer recovered their body condition. Shrubs and other vegetation on the forest floor of the island declined because of deer browsing pressure [27], and fallen leaves became an alternate and primary food resource for the deer throughout the year [28,46]. Thus, deer were exposed to severe food limitations [25], resulting in the decline of their body mass [25,47]. Body mass is one of the indicators representing the density-dependent change in deer body condition [6,48]. After the mass mortality event in the winter of 2003–2004, it was confirmed that female deer body mass slightly recovered compared to that recorded in the past several years [47], probably due to a large reduction in population density. Thus, improved deer movement across each block during the drive count, which was related to recovered deer body condition, may have occurred after 2004.

In the present study, observers were positioned on the lake in two small outboard-motor boats and at a vantage point on land to avoid double counting. In addition, we chose winter as the survey season because it provided improved visibility in the overstory and understory and permitted driving of wintering deer. Drive counts tracked temporal changes in deer abundance on the island; however, the difference between the drive counts and MR estimates changed temporally depending on density. Moreover, we confirmed a large uncertainty in drive counts due to unfavorable factors such as bad weather, despite the significant investment in time and personnel in the surveys. Furthermore, drive counts provided a point estimate only (they were not repeated in the present study as they were cost-prohibitive and laborious) and therefore, their precision could not be evaluated. Without precision evaluation, one cannot validate whether drive counts estimates reflect size variations in large populations. According to Dale and Beyeler [49], the ease of measuring is one of the important criteria that should be satisfied as an indicator of ecological change. Morellet et al. [6] suggested that wildlife managers invest resources in collecting additional data related to the present ecological status, rather than trying to estimate the absolute abundance index; hence, laborious drive counts may be directly opposed to such ideas, even if this method is used on steep and bushy forest areas. The block counts method [50] is also widely and conventionally used in Japan. However, Largo et al. [3] indicated that block counts often underestimate the population size of ibex (*Capra ibex*), even in alpine areas where there is high visibility. An additional limitation of block counts is reduced animal detectability derived from highly heterogeneous habitats [5]. If the usefulness of the abundance index is biologically uncertain, its continued use is a waste of resources, and the development and application of alternative methods should be considered [14]. Therefore, according to previous studies and our results, the use of conventional count methods (i.e., drive counts and block counts) and the monitoring effort for allocation of abundance indices on deer management in Japan may need to be reconsidered. Otherwise, if the drive counts method is used as an abundance index, Bayesian state-space model [51], which incorporates observation error into the estimation and integrates it into other abundance indices, may potentially work well.

In conclusion, we evaluated the possibility of an inherent risk of overestimation in drive counts by interpreting the results in accordance with an indicator of deer performance. Thus, to monitor large herbivore population-habitat systems, it is undoubtedly important that wildlife managers use a set of indicators of ecological change [6] instead of relying on absolute population sizes estimated by only one method.
